# Health teachers’ ethical conflict experiences in the COVID-19 situation: a qualitative content analysis

**DOI:** 10.3389/fpubh.2023.1265589

**Published:** 2024-01-05

**Authors:** Kyoung Mi Lim, Sohyune Sok

**Affiliations:** ^1^Department of Nursing, Graduate School, Kyung Hee University, Seoul, Republic of Korea; ^2^College of Nursing Science, Kyung Hee University, Seoul, Republic of Korea

**Keywords:** health teacher, ethical conflict, COVID-19, content analysis—qualitative, experience

## Abstract

**Background:**

In the context of the COVID-19 pandemic, health teachers who are responsible for the health of school staff and students are experiencing many ethical conflicts, and research on this is needed.

**Objective:**

This study was to investigate and explore the ethical conflicts experienced by health teachers during the COVID-19 pandemic situation.

**Methods:**

This was a qualitive study using directed content analysis applied to the four principles of biomedical ethics. Study participants were a total of 26 health teachers in Seoul, South Korea. In-depth individual interviews were conducted with 14 health teachers, and focus group interviews were conducted with the other 12 (2 teams with each 6 persons). Data were collected between May–June 2022, and analyzed using a deductive approach among the qualitative content analysis of Elo and Kyngäs. This study satisfied the four aspects of credibility, transferability, dependability, and confirmability presented by Guba and Lincoln (1989) to secure the reliability of qualitative research.

**Results:**

The ethical conflicts related to the four principles of biomedical ethics advocated by Beauchamp and Childress (autonomy, non-maleficence, beneficence, and justice), and the ethical conflicts in which overlap with the two principles of autonomy and non-maleficence, and the ethical conflicts related to miscellaneous matters or relationships, which were not included in the four principles, were investigated as the main 6 categories of ethical conflicts experienced by health teachers. Based on this, 10 generic categories and 17 subcategories were derived.

**Conclusion:**

This study could be used as primary data for policy development and intervention research. Such engagements can help identify ethical conflicts faced by health teachers in infectious disease crises, thus improving their ability to cope.

## Introduction

Since schools are densely populated with young students and faculty, there is a high risk of COVID-19 spreading quickly and widely in the event of an outbreak, which can impair students’ health and right to study; thus, systematic management is required. It is essential to take systematic measures to prevent the spread of infectious diseases by detecting them early and responding quickly (1). However, schools have not been able to respond quickly to the rapidly changing COVID-19 spread pattern, and the resulting variables, such as postponement of school opening, care gaps, and remote classes have relied on the discretion of health teachers and school principals to respond to the COVID-19 infectious disease, which caused a lot of confusion (2). As a result, the students’ and teachers’ physical and mental health were threatened. The health teachers responding at the forefront of infectious disease management in schools were reported to be significantly negatively affected due to the explosive increase in infectious disease-related work intensity and daily work paralysis (3). In addition, many health teachers were doing their best with a passionate and rational attitude to keep students and faculty safe from COVID-19, but they were faced with situations where it was difficult to make ethically appropriate judgments, and health authorities did not recognize the health teachers’ role for the prevention and control of COVID-19 in the early stages of the outbreak. Hence, they experienced ethical conflicts and suffering (4, 5).

According to previous literature that identified the level of ethical conflicts experienced by 307 health teachers in North Carolina, USA, 97.3% of health teachers were found to have experienced ethical conflicts (6). This figure is at the pre-COVID-19 level, and it is pointed out that the level of ethical conflicts among health teachers has increased due to COVID-19 (4). Health teachers experienced ethical conflicts through constantly changing standards of care, limited resources, lack of information on the COVID-19 spread situation in schools, and unfamiliar changes in work environments in the pandemic situation, leading to physical and mental problems such as frustration, guilt, anger, palpitations, insomnia, fatigue, withdrawal, and emotional fatigue (7). In addition, health teachers reported experiencing ethical conflicts due to their responsibility and the consequences occurring from not fully understanding the COVID-19 situation as a health teacher and not recognizing the complex health needs of students and fear of COVID-19 transmission among students, faculty, and their families, etc. (7). Accordingly, previous studies argue that the ethical conflict situation of health teachers should be explored more clearly, and that appropriate measures to resolve it should be discussed.

In South Korea, in a grounded theory study on the experience and role of health teachers in responding to infectious diseases (3), it was found that health teachers experienced anguish over the response as they faced endless and suffocating situations. However, this is a subcategory of ‘lonely struggling,’ which appeared as a central phenomenon in coping with infectious diseases of health teachers, and it was not enough to identify it as a situation of ethical agony or conflict. Additionally, there was only one paper (8) on the experience of clinical nurses’ bioethical dilemma in the COVID-19 situation. Looking at the studies on new infectious diseases published in South Korea (9–12), there are only studies on the experiences of health teachers in the situation of emerging new infectious diseases, and it was hard to find studies dealing with the ethical conflict of health teachers when responding to infectious diseases. Therefore, it would be meaningful to explore health teachers’ ethical conflict experiences in the COVID-19 situation.

Modern healthcare society faces numerous ethical problems with the development of medicine (13). In response, Beauchamp and Childress, in their book ‘Principles of Biomedical Ethics,’ explain biomedical ethics issues with the principle of respect for autonomy, the principle of nonmaleficence, the principle of beneficence, and the principle of justice (14). Elo and Kyngäs (15) mentioned that the deductive content analysis method can be used when verifying the explanation of an already theorized phenomenon and verifying it in a new context. In particular, Hsieh and Shannon suggested that the directed content analysis method can be used to confirm and expand the existing classification framework (16).

Therefore, based on the principles of biomedical ethics presented by Beauchamp and Childress, this study intends to explore health teachers’ ethical issues experienced in school health during the COVID-19 outbreak. This research, which has not been studied to date, was conducted through the directed content analysis method and aims to provide fundamental data for policy development and intervention research to improve health teachers’ ability to cope with ethical conflict situations.

In this regard, this study aimed to explore the ethical conflict situations experienced by health teachers in the COVID-19 situation and provide fundamental data for policy development and intervention research to improve the ability of health teachers to cope with ethical conflicts in an infectious disease crisis.

## Research questions

The research questions to find out the meaning and nature of the ethical conflict experienced by health teachers in the COVID-19 situation and to reveal deeper and more vivid experiences and meanings are as follows:

- What experiences did you have as a health teacher when COVID-19-confirmed cases occurred on campus?
- What ethical conflicts did you experience as a health teacher during the COVID-19 pandemic?

## Methods

### Study design

This study is a qualitative study that applied a deductive approach, the directive content analysis method, to explore the ethical conflict experienced by health teachers in the COVID-19 situation.

### Study subjects

Participants in this study consisted of 26 health teachers with more than 1 year of experience who had experience in responding to COVID-19 outbreaks and were willing to participate in the study (Table 1). Of the 26 health teachers, 14 participated in individual in-depth interviews, and 12 health teachers participated in the focus group interview conducted to elicit in-depth thoughts or opinions through free discussion.

**Table 1:** General characteristics of the study participants (*N* = 26).

Item	Gender	Age (year)	Marital status	Place of work	School type	Teacher careers (month)	Frequency of interview
**Individual interview**							
1	Female	42	Married	Elementary	Private	52	3
2	Female	32	Married	Elementary	National	30	1
3	Female	34	Single	Elementary	National	36	2
4	Female	31	Married	Elementary	National	68	1
5	Male	32	Single	Middle	National	30	2
6	Female	31	Married	Elementary	National	43	1
7	Female	30	Single	Elementary	National	24	2
8	Female	32	Single	High	National	84	1
9	Female	35	Single	High	National	30	1
10	Female	32	Single	Middle	National	34	1
11	Female	36	Married	Middle	National	82	1
12	Female	28	Single	Elementary	National	33	1
13	Female	41	Married	Elementary	National	94	2
14	Female	35	Married	Middle	National	108	3
**Focus group interview**							
1	Female	30	Single	Middle	National	40	1
	Female	32	Single	High	National	40	
	Female	27	Single	Middle	National	40	
	Female	46	Married	Middle	National	43	
	Female	30	Single	Middle	National	50	
	Female	32	Single	Elementary	National	60	
2	Female	54	Single	Elementary	National	280	1
	Female	55	Married	Elementary	National	394	
	Female	48	Married	High	Private	222	
	Female	45	Married	Elementary	Private	149	
	Female	44	Married	Middle	National	108	
	Female	56	Married	Elementary	National	408	

### Data collection

Data on health teachers’ ethical conflict experiences in the COVID-19 outbreak were collected through individual in-depth interviews and focus group interviews. Individual in-depth interviews with 14 study participants were conducted from May 13 to June 30, 2022. All interviews were conducted at the time and place desired by the participants and were recorded after obtaining prior consent. Each participant was interviewed at least one to a maximum of three times, and additional interviews were conducted if it was necessary to confirm the contents during data analysis. The main questions used in the interviews were prepared after the researcher determined the contents of the questions through a literature review in advance and clarified the study’s objectives. Afterward, with the advice of two nursing college professors, a semi-structured open-ended questionnaire and interview guidelines were completed. The questionnaire consisted of opening, introductory, main, and closing questions. The main question was “What ethical conflict situations did you have as a health teacher during the COVID-19 outbreak situation?” to explore the essence of what the subjects said more deeply by having them explain specific cases or specific situations for what they specially experienced because they were health teachers.

Focus group interviews were conducted group by group for 2 days, June 20 and 28, 2022, after discussing the location, date, and time of the meeting where all the study participants could gather. For a more efficient focus group interview, the research methodology and the questions’ form and principle were reviewed after consultation with two nursing college professors and the previous literature on the focus group interview methodology (17, 18). The questionnaire consisted of opening, introductory, main, and closing questions, the same as the individual in-depth interview. The main question was the in each case, “What ethical conflict situations did you have as a health teacher during the COVID-19 outbreak situation?” Additionally, questions connected to the main question were added as needed to check the saturation of focus group interviews. Some questions were as follows: “If there is someone who has had a different experience than what was said previously, please tell us” and “What is the opinion of others? Were there any unexpected situations?” Each focus group interview was conducted for about 3 h per group, and the subjects were allowed to talk freely, but all subjects spoke at least once so that all subjects could participate.

In qualitative research, it is important to reach data saturation for a sufficient and rich description of a phenomenon (18). In this study, no new data appeared after individual in-depth interviews with 14 health teachers and two focus group interviews, so it was judged that the data had reached saturation.

### Securing the quality of research results

This study intended to satisfy the four aspects of credibility, transferability, dependability, and confirmability presented by Guba and Lincoln (19) to secure the reliability of qualitative research. First, to ensure credibility and show how accurately the research results describe and interpret the actual phenomenon, this study strived to select participants who could well express the ethical conflict experiences of health teachers during the COVID-19 outbreak. To this end, the study’s objective was explained before each interview with health teachers who expressed their desire to talk about their vivid experiences related to the topic. During the interview, notes were made on the situation at the time, including the key contents and behavior of the participants, and the researcher himself/herself transcribed them quickly after the interview to ensure that there were no data omissions or distortions. In the analysis, uncertain parts, parts where the meaning was not clearly revealed, or ambiguous expressions were confirmed directly with the participants through phone calls, messages, or e-mails. Second, as to whether the research results could be applied to other situations, the researcher confirmed the saturation of the contents while collecting data and performing analysis simultaneously so that the study’s findings could have generality and representativeness. The transferability of the research results was verified by showing and confirming the research results with two health teachers other than the participants and one nursing college professor with experience as a health teacher. Third, to secure dependability, this study used an audit trail. Dependability was verified and validated by experts—two professors with experience in qualitative research. Consequently, study participants who experienced ethical conflicts during the COVID-19 outbreak situation were selected, the appropriate contents of the questionnaires for individual in-depth interviews and focus group interviews were drafted, data collection and analysis were properly performed, the data saturation checking was checked, and the research results were reviewed and evaluated to determine if they met research objectives. Fourth, this study used an audit trail to ensure confirmability. To do this, first, all possible information on the data related to the study, such as the time of data collection, the contents of behavior, and the outcomes, was included through the researcher’s daily log and recording device. Second, through regular meetings with two nursing college professors with experience in qualitative research and other data were shared to complete the data analysis; hence, the researcher’s individual subjectivity and prejudice were excluded.

### Ethical considerations

This study was conducted after receiving approval from the Institutional Review Board of K University (Approval No. XX). The researcher explained the purpose of this study face-to-face to health teachers who expressed their intention to participate in the study, provided an explanatory statement, and allowed them to voluntarily decide whether or not to participate in the study. The recording about the purpose of the study, the progress of the interview, key questions during the interview, the duration of the interview, and the contents of the interview were explained to the study participants before the interview begin. It was also explained that the recorded files and records would only be viewed by the researcher. For privacy protection, it was explained that the recorded contents and transcripts would be stored on the researcher’s personal PC and external hard drive using a password. In addition, it was explained that the collected data would be destroyed using a document shredder after being stored for 3 years, and the recorded contents would be deleted. It was explained that the recorded contents would not be used for any purpose other than the study, and that participation in the study could be discontinued at any time during the study and that there would be no disadvantages to the participant.

### Data analysis

In this study, the data obtained through individual in-depth interviews and focus group interviews were analyzed using a deductive approach among the qualitative content analysis of Elo and Kyngäs (15). Deductive content analysis is useful when verifying in a new context based on existing theories, and of the method, directed content analysis can be helpful when existing theories or previous studies are incomplete or require additional explanation for existing phenomena (16). In this study, directed content analysis was adopted as a deductive approach to verify the ethical conflict experience of health teachers by applying the existing four principles of biomedical ethics and to discover other ethical conflict situations. To perform deductive content analysis, first, a categorization matrix must be created and coded according to the data category. Therefore, in this study, the four principles of biomedical ethics proposed by Beauchamp and Childress, the principle of respect for autonomy, the principle of nonmaleficence, the principle of beneficence, and the principle of justice, were set as a categorization matrix. Second, while coding with the four principles as the main category, overlapping contents between the principles were classified as a separate main category. In addition, contents not included in the four principles were also classified as a separate main category. Finally, similar sentences and paragraphs were conceptualized and subdivided into general categories and subcategories within the main category.

Data analysis was conducted immediately after the data collection was completed in order to vividly organize the opinions of the participants after the interview. The researcher transcribed all of the audio-recorded interviews, prepared them as transcripts, read the transcripts over several times, and at the same time conducted line-by-line analysis keeping in mind the main concepts and meanings.

## Results

### Content analysis for health teachers’ ethical conflict experiences during the COVID-19 outbreak

As a result of analyzing the contents of individual in-depth interviews and focus group interviews conducted to explore health teachers’ ethical conflict experiences in the COVID-19 situation, a total of 307 meaningful statements were found. The four principles of biomedical ethics (principle of respect for autonomy, principle of non-maleficence, principle of beneficence, principle of justice) presented by Beauchamp and Childress, and the conflict situations in which the two principles overlap (principle of respect for autonomy and principle of non-maleficence) and the conflict situations related to other relationships, these six main categories were explored as main categories of ethical conflict experienced by health teachers in the COVID-19 situation. Based on this, 10 generic categories and 17 sub categories were derived. Table 2 shows the details of ethical conflict situations for each major category.

**Table 2:** Content analysis for health teachers’ ethical conflict experiences during the COVID-19 outbreak.

Main category	General category	Subcategory
Ethical conflicts related to the principle of respect for autonomy	Agony between respect for the right to self-determination and the responsibility of health teachers	Agony between vaccination based on autonomous decision-making and vaccination for herd immunity
		The conflict between the action of returning home for those with suspected symptoms and violating the right to learning
Ethical conflicts related to the principle of nonmaleficence	Pressure to make choices that do not harm all school members	Agony about finding the best choice that is not harmful in various situations that are not in the manual
		Anxious about non-face-to-face classes for the right to health and the care of students who are left alone at home during non-face-to-face classes
Ethical conflicts related to the principle of beneficence	The feeling of loss for not providing sufficient care to school members	Situations where sufficient care cannot be provided to students and faculty who need psychological support due to overwork
		Concerns about gaps in daily school health work due to increased work related to COVID-19
		Situations where active measures should be taken to prevent the spread of COVID-19, but passive measures are taken out of concern that my family and I will contract COVID-19
		Situations where due to the perception that only health teachers are responsible for COVID-19-related work, health teachers are burned out as heavy work continues, and as a result, it is impossible to provide care with good capacity
Ethical conflicts related to the principle of justice	The feeling of helplessness in the unfair distribution of limited medical resources	Hostility to the circumstances of prioritization of superiors’ school health services related to COVID-19
	The feeling of deprivation due to discrimination in the criteria, such as achievements, efforts, etc.	Difficulty performing the best work due to the lowered self-esteem resulting from discriminatory compensation
		Difficulty performing the best work due to lowered sense of duty resulting from not being recognized
Conflict experiences where two principles overlap (respect for autonomy and nonmaleficence)	The dilemma between privacy protection and information sharing	Concerns between information sharing and privacy protection for groups vulnerable to COVID-19 infection
	Situations where illegal acts must be endured to prevent the spread of infectious diseases	Heavy-hearted for collecting personal information of students and faculty in advance to prevent the spread of COVID-19
		Situations where there is no choice but to share personal information and travel routes with health teachers to prevent the spread of COVID-19
Others (conflict experiences related to relationships)	Agony between conflicts with family and duties of health teachers	In the prolonged COVID-19 situation, conflicts with families prevented health teachers from performing their duties
	Doubts about the value of the health teacher’s job	Skepticism about the situation where the professional opinions of health teachers were swayed by the unprofessional opinions of school administrators
	Insufficient cooperation system with related organizations	Pessimistic about the situation where related organizations and frontline schools are sympathetic to the defensive attitude to avoid taking responsibility

### Ethical conflict experiences of health teachers related to the principle of respect for autonomy

#### Agony between respect for the right to self-determination and the responsibility of health teachers

In the COVID-19 situation, health teachers knew they needed to deliver accurate information about vaccine safety and side effects to students and faculty and allow them to choose whether or not to get vaccinated based on their autonomous decision-making. However, it was identified that they experienced ethical conflicts in the situation where they had to encourage students, faculty, and their families to vaccinate against COVID-19 in accordance with the Ministry of Education’s recommendation for preventive vaccination to prevent the spread of COVID-19.

“Serious side effects are being reported, and even I have anxiety about the vaccine, but I have to encourage children and their parents to get vaccinated. (Omitted)… That is the part that we cannot force, but from the health teacher’s point of view, we cannot just leave them to their own discretion.” (Teacher 2).

In addition, it was found that health teachers experienced ethical conflicts when parents refused to send students home because the right to learning was violated when health teachers took action to send home suspected patients who were not confirmed COVID-19 cases.

Some parents even say, “If my child is not a confirmed case, how are you going to take responsibility? How are you going to take responsibility for missing classes?”…then I’m hesitant to make a decision. If the student is not a confirmed case, and the symptoms are mild, it becomes even more difficult to make a decision.” (Teacher 10).

### Ethical conflict experiences of health teachers related to the principle of nonmaleficence

#### Pressure to make choices that do not harm all school members

During the COVID-19 outbreak, health teachers experienced ethical conflicts about decision-making not to harm students and faculty, considering that they face numerous variables that could not be found even in their manual.

“The moment-by-moment decisions are all our responsibility, and the school asks me to express my opinion because I am an expert… (omitted) In fact, this situation is something we have never experienced… (omitted)… So, I was afraid at every moment if the situation became bad due to my shortcomings.” (Teacher 1).

In addition, when health teachers tried to change the teaching method to non-face-to-face classes to prevent COVID-19 transmission, it was determined that they experienced a series of conflicts when making decisions because there is a high probability of a care gap, such as students from various types of vulnerable families being left alone at home due to non-face-to-face classes.

“I’m trying to adopt non-face-to-face classes because they are safer, but for children from vulnerable families, money, the financial issue, is the number one priority right now! (omitted) As a result, the children are left alone at home. So, I kept conflicting, thinking if I keep emphasizing only health, is not it pushing children out of school? Is it best to choose now?” (Focus Group Interview 1).

### Ethical conflict experiences of health teachers related to the principle of beneficence

#### The feeling of loss for not providing sufficient care to school members

First, students diagnosed with the COVID-19 had to spend a difficult time due to the problem of stigma in the early stages, and health teachers experienced ethical conflicts—they were unable to provide the best care to the students due to overwork.

“The first confirmed student has yet to come to school. So, it’s been 3 months, and the fact there is someone who has not come back yet… (omitted)… (Researcher: What kind of trauma was that? Psychological trauma?) I think 100% that’s it. But there is nothing I can do for this student. I am still very busy with other schoolwork. Am I doing my job properly as a health teacher?” (Teacher 1).

Second, the health teachers experienced ethical conflicts as they were frequently out of the health room due to the increased work related to COVID-19 quarantine.

“With more work and more health education classes due to the corona, the need to be out of the health room is increasing, so the probability of missing a patient is increasing. (omitted) Corona keeps creating a vacuum in the work of health teachers. But I cannot stop working, and on the contrary, I cannot just guard the health room, and it’s really embarrassing.” (Teacher 7).

Third, the health teachers feared the possibility of being infected and affecting their families and the students visiting the health room in case of a direct contact with a confirmed COVID-19 patient. Indeed, the health teachers experienced ethical conflicts during such a situation; hence, they treated students passively.

“I am afraid that I will be confirmed, and more than that, I am afraid that I will become infected and spread the infection while caring for students, and I am more afraid that I will pass it on to my family. As a result, until the parents arrive, I leave the child in the observation room and watch from the outside. My heart wants to be close to the student, but on the other hand, I cannot.” (Teacher 8).

Fourth, due to the perception that responding to COVID-19 is the job of only the health teacher, health teachers experienced ethical conflicts when they had to take on various administrative tasks, leading to physical and mental exhaustion due to work overload. Consequently, they were unable to provide the best care all the time as the ones responsible for taking care of students’ and faculty’s health.

“Since I continued almost 80 to 90% of the infectious disease control by myself, it was very difficult for one person to do it in the end, because when it comes to corona, it all comes to the health teacher. (omitted)… I was eager to put everything down. After that, there came a moment when I felt that even students and faculty members coming to the health room were annoying.” (Teacher 10).

### Ethical conflict experiences of health teachers related to the principle of justice

#### The feeling of helplessness in unfair distribution of limited medical resources

The health teachers experienced ethical conflicts because of the unfair distribution of limited medical resources during the COVID-19 outbreak.

“Cannot you just put a sample for my daughter? I’m getting a test here, and you can just hold the hands of my daughter like this and do it!… (omitted)… But it’s really not easy to cut and say no. “cause good is good, but it always bothers me.” (Teacher 3).

#### Feeling of deprivation due to discrimination in the criteria, such as achievements, efforts, etc.

It was found that health teachers experienced ethical conflicts when they saw themselves changing from active work attitudes to passive work attitudes as their self-esteem plummeted due to differential compensation for achievements related to responding to COVID-19.

“Public health officials and health teachers are actually doing the same thing, but public health officials have compensation for their work and things like that, but we do not have any of those things, even though we are the same public servants… (omitted)… I feel like my self-esteem is going down. Then, I think, I do not have to work so hard! When we feel that way, it becomes very difficult to work.” (Focus Group Interview 1).

In addition, it was found that the sense of duty of the health teachers decreased as they faced a situation where the efforts to protect school members from the threat of the COVID-19 pandemic were regarded as a natural task of the health teachers, leaving the health teachers’ efforts and labors unrecognized. As a result, the health teachers perceive that they cannot do their best in their responsibilities as health teachers.

“Health teacher! Have you ever been here when more than 100 calls were made to the teacher’s office? He said, “what did you do in the health room?” I was dumbfounded, so I said I contacted the Office of Education, parents, homeroom teachers, and the public health center to proceed with the work. I’ve never had a break… (omitted) … So, as long as there are no major problems, there will be no turmoil. Then, let us just do that, let us just endure it like that. (Focus Group Interview 2).

### Ethical conflict experiences where two principles (respect for autonomy and nonmaleficence) overlap

#### Dilemma between privacy protection and information sharing

In a situation where health teachers were aware of the urgent need to share information about confirmed cases for the health of high-risk populations such as those with allergic asthma or leukemia who are vulnerable to COVID-19 infection, but active cooperation was not possible without an approval process according to the personal information protection principle, health teachers experienced ethical conflicts appearing as the two principles (respect for autonomy and nonmaleficence) overlapped.

“Because of the thought that friends already know where the student lives… (omitted)… I wonder if I should tell them where the student lives… A mother with a child who suffered from congenital heart disease was even more sensitive. She worried a lot if her child might be in the same class or the traffic lines overlapped, but I did not tell her after all.” (Teacher 6).

### Situations where illegal acts must be endured to prevent the spread of infectious diseases

In collecting and utilizing the personal information of COVID-19-confirmed patients, health teachers were forced to endure illegal acts, such as collecting or sharing personal information in advance without approval procedures to prevent the spread of infectious diseases effectively. As a result, health teachers experienced ethical conflicts as the two principles (respect for autonomy and nonmaleficence) overlapped.

“When a confirmed case occurs, we have to look at the Nice health record and figure out personal information. But it’s a waste of time for me “cause we need to contact quickly to stop the spread. (omitted)… I do it because I have to do it in advance so that when a confirmed case comes out, I can proceed quickly and prevent the spread of infectious diseases more quickly, so I do it, but because it should not be done like this, conflict arises.” (Teacher 3).

“In violation of the Personal Information Protection Act, health teachers are sharing information about confirmed cases. (omitted)… If you do not have accurate information to take any action with the story you just heard, there will be setbacks in class operation at school. We pay the fine. Without it (sharing information in the health teacher community), I cannot do my job. (omitted)… Even among health teachers, there are many concerns about what we have to work (with) even while breaking the law like this.” (Teacher 8).

### Others (ethical conflict experiences related to relationships)

In the COVID-19 outbreak, health teachers were experiencing ethical conflicts even in situations not classified under the category of the four principles of biomedical ethics.

### Agony between conflicts with family and duties of health teachers

As the COVID-19 situation has been prolonged, there have been frequent cases of causing inconvenience among family members or hearing resentment from family members. Thus, the health teachers experienced ethical conflicts, such as a case where conflicts with the family have led to a passive attitude as a school health manager, guilt, and hesitation.

“Calls came from school again and again, and I was having a hard time trying to answer the phone, so my mother-in-law said, “Cannot you just not answer it once? If they are in a hurry, they will do it one more time!” I was so sad when she said that. (omitted)… So, “do you want me to not answer the phone once? This is what I have to do!” I just walked out while saying it. (omitted)… I was so sorry. Before being a health teacher, I am a daughter-in-law and family member, and I was pathetic because I prioritized schoolwork over my family work, and on the other hand, I was upset because my family did not seem to understand, and I felt various complicated feelings.” (Teacher 8).

### Doubts about the value of the health teacher’s job

In response to the COVID-19 infectious disease, the health teachers experienced ethical conflicts, feeling relieved at the situation of putting down heavy responsibilities when unprofessional opinions directed by school administrators swayed the professional opinions of health teachers.

“My school principal’s philosophy of education is that children should come to school and eat. He has such (a) philosophy… (omitted)… I think we still have to do more remote classes, but they do not even listen to me even though I talk here anyway… so I need to get out of here. Otherwise, I’ll just be burned out. But it’s not my responsibility… I thought about this a lot.” (Focus Group Interview 1).

### Insufficient cooperation system with related organizations

The health teachers experienced ethical conflicts when they saw related organizations and schools showing a defensive attitude not to take responsibility for COVID-19 quarantine activities, and the health teachers themselves agreed to it.

“They said “Isn’t it of course necessary to discuss with the public health center what to do with exceptions? (omitted)… If you are worried because they are students, you should discuss it with the Office of Education.” What are you talking about right now… Why does the Office of Education stop them (from) going to school, what does it know… We toss to each other while putting it off, then it is tossed back to school. (omitted)… The manual says to discuss this, but when we discuss it, they say like why are you asking us… They put it off because they do not want to take responsibility, and I’m doing the same… I just do not want to do it.” (Teacher 4).

### Reflection through crisis response in the COVID-19 outbreak situation

The health teachers have been in charge of general duties related to infectious diseases in schools, such as examination, epidemiological investigation, treatment, quarantine, prevention education, official document processing, and temporary observation room management. In the early days of the COVID-19 outbreak, it was found that they had a difficult time due to guilt and criticism. They were also gradually burned out while suffering from the heavy responsibility to prevent the spread in schools and handling excessive work every day. Through work division, they tried to put down the burden on their mind and recover their exhausted body and mind to find their true self. Psychological changes were studied, such as a positive and grateful attitude for trivial daily life while going through and overcoming the big thing called COVID-19 and an increase in empowerment by gaining confidence in management even if another infectious disease appears.

“Since I am said to be responsible for this, I feel like everyone knows me, and the thought “what will they think of me?” made me shrink a lot. Because of the thought, the work shrinked (shrank)… That was the biggest change in my daily life.” (Teacher 1).

“At first, I did not have time to think about myself… I mean, because I had so many things to do… I was so busy taking care of this, without knowing the time was passing, so I ended up getting off work at 8 or 9 every day, and tears came out strangely on the bus. (Focus Group Interview 1).

“I think letting it go helps. So, I cannot fully respond to everything… (omitted)… I tried to find my real self with me working as a health teacher by making something dissociate. (Focus Group Interview 1).

“Even though it was really difficult at the time, I am grateful. After going through hard times at that time, after a while, it became my sole asset… (omitted)… After going through hard times once, I became stronger and honestly, I gained trust in myself. (omitted)… There will be many junior health teachers looking at me one day… Wouldn’t I be an advisor who can guide them? Because of that, I always try not to lose my gratitude.” (Teacher 8).

## Discussion

Among the four principles of biomedical ethics presented by Beauchamp and Childress, the ethical conflict experienced by health teachers concerning the first principle of respect for autonomy was the conflict between vaccination based on autonomous decision-making and vaccination for herd immunity. Health teachers should advise all school members to be vaccinated to protect students and faculty from COVID-19, according to the policies of the government and the Ministry of Education. However, the vaccine’s safety was not assured, and even the health teachers themselves were reluctant to be vaccinated due to anxiety about the vaccine; thus, recommending vaccination in such a situation was found to cause a great psychological conflict. It is considered that health teachers must have experienced an ethical conflict over the need to encourage vaccination rather than the wishful choice of students and faculty in a situation where there is no firm belief in the vaccine’s safety since it was developed in just 8–9 months (20, 21). Moreover, it is believed that new guidelines and policies compatible with the ethical value of respect for life and personal autonomy must be developed. Hence, the health authorities and the Ministry of Education must prepare and provide sufficient information about vaccines and guidelines on its side effects before vaccination, training, and educational materials for health teachers to establish a system that enables health teachers to escape ethical conflicts about vaccines.

Among the principles of biomedical ethics, health teachers experienced ethical conflict in relation to the second principle of nonmaleficence while facing situations where they had to make choices that did not harm all school members. Health teachers were found to be in a conflicting situation concerning if the decision that was made by them alone, the only medical staff in the school, caused harm to students or faculty because even though the government has revised the COVID-19 response guidelines and manuals several times and distributed them to schools, there were still many situations that were not in the guidelines and manuals. Considering the work environment of a health teacher who works alone in a health room, practical case-based manuals or education are essential in a situation where the best decision must be made. In addition, health teachers experienced a difficult ethical decision in the situation where they could not take care of students suspected of infection at school to guarantee the right to health of other students, even though it is highly likely to have a care gap when returning students suspected of COVID-19 infection to their homes due to the lack of understanding of school infectious disease control by parents who have to devote themselves to livelihood to address their economic difficulties. In a crisis such as the COVID-19 pandemic, it is urgent to establish a systematic institutional support system linked to school health to prevent a care gap for children, and it is judged that infectious disease control education for guardians should be actively carried out.

Among the principles of biomedical ethics, health teachers experienced ethical conflict about the third principle of beneficence, arising from insufficient care to school members for reasons such as work overload during the COVID-19 outbreak, which could result in risking the health teachers’ and their families’ safety. First, it was found that health teachers experienced ethical conflicts when they could not provide sufficient care to students and faculty stigmatized by COVID-19 confirmation. Although health teachers were seriously aware of the stigma related to infectious diseases (22, 23), due to lack of time caused by overwork, health teachers were unable to actively intervene and mediate students’ mental health problems, which led to an ethical conflict. Therefore, it is necessary to develop activities that aim to increase social awareness of stigma related to school infectious diseases and provide specific guidelines on how to respond to and prevent stigma. Second, health teachers experienced ethical conflicts when they were frequently out of the health room due to increased COVID-19-related work, resulting in a work vacuum, making it impossible to respond to emergencies of students or staff. It is thought that a regulation including a disaster situation is necessary for preparing regulations on the delegation of nursing duties by health teachers in preparation for emergencies (24, 25). Third, health teachers experienced ethical conflicts as COVID-19 acted as a hindering factor for them to do their best in performing their duties—they were afraid of the possibility of infection by contact with confirmed COVID-19 patients and responding with a passive attitude rather than responding best to students confirmed with COVID-19. The ethical conflict that health teachers feel between personal safety and responsibility as a health teacher is directly related to the safety of students and faculty, so it is necessary to continuously expand quarantine personnel and establish a more detailed care process for confirmed students and faculty members in schools as a way to solve this problem. Fourth, health teachers experienced the ethical conflict of wanting to get out of the responsibility as a health teacher and return to daily life for themselves. They were physically and mentally exhausted due to heavy work during the COVID-19 pandemic, and they experienced difficulties in daily life and reduced work efficiency. Due to the perception of faculty members that all tasks previously performed by other faculty members were handed over to health teachers when the word COVID-19 was added, health teachers performed heavy work every day and were burned out inevitably (3, 12). Consequently, it is thought that the health teachers felt frustrated and resentful when they could not provide the best care for students and faculty. It is also believed that ethical conflicts caused by health teachers’ work overload can be resolved as school members are fully aware of their roles and health teachers and school members effectively communicate in a disaster situation such as COVID-19.

Among the principles of biomedical ethics, health teachers experienced ethical conflict in relation to the fourth principle of justice, arising from experiencing the unfair distribution process of limited medical resources in the COVID-19 situation and discrimination in the criteria such as achievements and efforts. Health teachers are required to provide school health services fairly and equitably to students and faculty during the COVID-19 outbreak, but when a superior requested them to provide school health services first, they had to accept it without resolute refusal, according to the principle, which led to an ethical conflict. To efficiently respond to novel infectious diseases that have become prevalent as a pandemic, such as COVID-19, it is very important to distribute medical resources such as school health services fairly and equitably. Therefore, it is assumed that ethics education is essential in infectious disease control for school administrators, including health teachers. Additionally, in terms of ethical conflicts based on discrimination in the criteria such as achievements, efforts, etc., health teachers experienced ethical conflicts as they found it difficult to perform their best work due to the lowered self-esteem resulting from discriminatory compensation and the lowered sense of duty resulting from not being recognized. Despite performing the same duties in response to COVID-19, health teachers experienced discrimination compared to other public health officials, such as the use of vacation and annual leave, payment of allowances, etc. (26), and their achievements and labors in responding to COVID-19 were not properly recognized by other faculty members. As a result, they experienced a reduced sense of achievement and satisfaction as a health teacher, which lowered their sense of duty. Hence, it is claimed that a systematic improvement plan should be prepared to ensure respect for achievements and reasonable performance-related pay by confirming the factors recognized by health teachers as discrimination in detail. The ethical conflict experienced by health teachers occurred while handling and utilizing personal information during a conflicting situation where two principles of biomedical ethics (respect for autonomy and nonmaleficence) overlapped. The conflict occurred as the principle of respect for autonomy, which requires approval and consent when sharing personal information by respecting the individual’s right to self-determination, and the principle of nonmaleficence, which requires minimizing the risk and pain inflicted on others by sharing confirmed patient information, overlapped. The study’s findings revealed that the health teachers experienced an ethical conflict between sharing information about confirmed COVID-19 patients for vulnerable populations, such as those with allergic asthma or leukemia, and protecting personal information. From the perspective of parents with high-risk children suffering from diseases that are susceptible to infection, it is vital to identify the traffic line of COVID-19-confirmed students and take measures to prevent their children from having the overlapping traffic line. For cases where reasons must be provided in a reasonable and timely manner, such as sharing personal information of confirmed cases for vulnerable populations to infectious diseases, it is necessary to revise policies or laws that limit the items and scope of personal information to be used and to have specific guidelines. In addition, health teachers faced a situation where they had no choice but to endure illegal acts to prevent spreading infectious disease. It was found that they experienced an ethical conflict when they were forced to collect the personal information of students and faculty in advance to prevent spreading COVID-19, even though it was illegal, or to share the personal information and traffic line of confirmed cases with the community. In a school health environment where many people can be infected simultaneously, using personal information to prevent the spread of infectious diseases is essential. Therefore, in preparation for future epidemics of infectious diseases, it is judged that the Ministry of Education should have a policy that includes guidance, educational materials, and information utilization standards for health teachers.

Conflicts that were not classified into the category of the four principles of biomedical ethics were grouped into other categories and identified as ethical conflict experiences related to relationships with family, school administrators, related organizations, etc. First, it was found that health teachers experienced ethical conflicts which they passively coped with when performing their duties as health teachers due to conflicts with their families in the prolonged COVID-19 situation. As the COVID-19 situation is prolonged, health teachers, as the last bastion of taking responsibility for school health, had a conflict with their families in a situation where the health of school members should be prioritized, which seemed to cause ethical conflicts such as having a passive attitude as a school health manager. If these conflicts with families persist, it will hinder the health teacher’s work immersion (8, 10). Ultimately, it is directly related to the health problems of school members, so it is necessary to prepare measures to solve the problem of conflicts with families that health teachers are experiencing. Therefore, in forming school organizational culture, it is considered that creating an organizational culture where families can be involved or constant interest and support of school administrators is necessary. Second, it was found that health teachers experienced ethical conflicts while facing a situation where people were swayed by the school administrator’s unprofessional opinion rather than the health teacher’s professional opinion. That said, school administrators want to operate the school as normally as possible as they consider parental complaints and school management issues first, so there are times when there is a difference in opinion with health teachers who think of quarantine first (3). It is thought that health teachers were fulfilling their responsibilities even in difficult situations with professionalism and a sense of duty, but they might have doubts about the value of their duties as health teachers, showing limitations such as obeying the instructions of school administrators rather than their opinions as school health experts. Indeed, it will be necessary to develop education and programs that can enhance the professionalism of health teachers to boost their sense of calling as health teachers. Third, health teachers were found to have experienced ethical conflicts when they were sympathetic to the defensive attitude of not taking responsibility between related organizations and front-line schools in the COVID-19 situation. They said that the Office of Education had not played the role of a control tower when responding to an infectious disease outbreak in schools and that the work cooperation system was insufficient, such as issuing guidelines to public health centers without prior consultation. They argued that it was difficult because the public health center adhered to the principles, did not respond to requests for cooperation, or was not involved in the school’s decision to close schools, avoiding responsibility (3). Discord between related organizations is a direct factor that lowers the quarantine capacity in the region, so it is an issue that needs to be addressed urgently. Therefore, it is significant to reestablish a strategic cooperation system by identifying the cause of the problem among the education authorities, health authorities, and front-line schools, which are not locally cooperated in the community, and by deriving improvement measures.

### Implications for practice, policy, and research

Health teachers need a high level of ethical sensitivity to recognize various ethical issues that arise during an infectious disease outbreak and make the best decision. Therefore, the research results can be used as a fundamental reference for future methodological research to identify the level of ethical sensitivity of health teachers during the COVID-19 outbreak and to solve the ethical conflict of health teachers through case studies related to the ethical dilemmas of health teachers. In addition, the research results can be used as evidence research data for establishing an institutional support system, such as organizing an ethics committee under the Ministry of Education, which can receive expert help for ethical conflict situations that occur in schools. The research results can be utilized to identify the factors that health teachers perceive as discrimination during the COVID-19 outbreak and to prepare systematic improvement plans to ensure reasonable performance-related pay.

Furthermore, the following measures are needed for substantial policy and institutional improvement. It is necessary to establish a systematic institutional support system linked to school health and prepare supplementary measures to prevent childcare gaps from occurring during health crises such as pandemics. Regulation is needed in preparing the delegation of health teachers’ nursing duties during emergencies. Specific guidelines related to personal information that may occur in school health should be presented to protect health teachers experiencing ethical conflicts related to personal information, and revision of policies or laws are required to prevent personal information exposure. New guidelines and policies on vaccination compatible with the ethical value of respect for life and personal autonomy must also be developed. Also, to resolve conflicts caused by health teachers’ lack of communication, it is necessary to seek expert opinions, prepare communication channels in advance to discuss school situations, and develop practical case-oriented manuals. Most importantly, to increase the capacity to respond to infectious diseases, it is important to reestablish a local cooperation system among related organizations (education authorities, health authorities, front-line schools, etc.) in the community.

### Limitations

This study had some limitation. Since this study targeted health teachers in some regions, there are limitations in generalizing the results of this study. However, exploring the ethical conflicts experienced by health teachers in the context of an infectious disease crisis is an important study in establishing an efficient school health system for infectious disease management and improving the ability of health teachers to respond.

## Conclusion

In conclusion, this study explored the ethical conflict experience of health teachers by substituting the four principles of biomedical ethics presented by Beauchamp and Childress in order to gain an in-depth understanding of the ethical conflict experienced by health teachers in the COVID-19 situation. Also, in order to discover other ethical conflicts, the method of directive content analysis was applied. As a result, 6 main categories, 10 general categories, and 17 sub categories were derived.

This study is meaningful because it can be used as fundamental data for policy development and intervention research to identify ethical conflicts experienced by health teachers during an infectious disease crisis and improve their coping skills. In addition, it is expected that the research results can be used as fundamental data for the development of training materials and manual improvement activities and assist the Ministry of Education or Office of Education, particularly the infectious disease policy managers in health authorities, in identifying the roles of health teachers and the ethical conflicts during an infectious disease crisis.

## Data availability statement

The raw data supporting the conclusions of this article will be made available by the authors, without undue reservation.

## Ethics statement

The studies involving humans were approved by Institutional Review Board of Kyung Hee University [approval number: KHSIRB-21-370 (RA), approval date: September 16, 2021]. The studies were conducted in accordance with the local legislation and institutional requirements. The participants provided their written informed consent to participate in this study. Written informed consent was obtained from the individual(s) for the publication of any potentially identifiable images or data included in this article.

## Author contributions

KL: Conceptualization, Data curation, Formal analysis, Investigation, Methodology, Validation, Visualization, Writing – original draft, Writing – review & editing. SS: Conceptualization, Data curation, Formal analysis, Investigation, Methodology, Resources, Validation, Visualization, Writing – original draft, Writing – review & editing.
